# Supplementary material for: Comprehensive Annotation and Functional Exploration of MicroRNAs in Lettuce
Source: Front Plant Sci. 2021 Dec 24;12:781836. doi: 10.3389/fpls.2021.781836 (PMC8739914; doi:10.3389/fpls.2021.781836)
Supplement: Supplementary file 2 [file Data_Sheet_8.ZIP › GSM2230747.plot/Lsa-miRN55_Lsat_1_v5_gn_9_11361.1_105_TPlot.pdf]

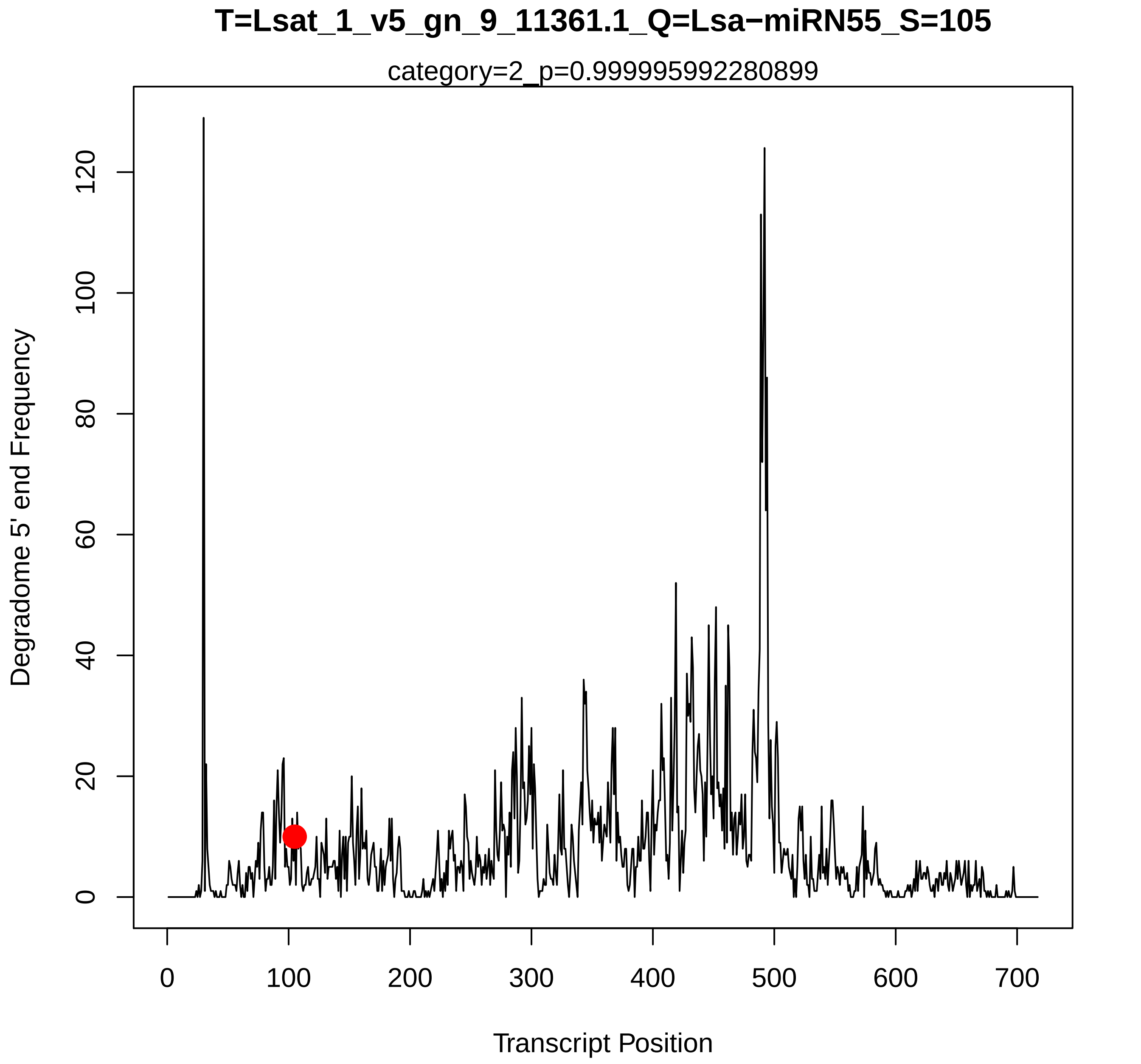
T=Lsat_1_v5_gn_9_11361.1_Q=Lsa−miRN55_S=105
category=2_p=0.999995992280899
Degradome 5' end Frequency
Transcript Position
0
20
40
60
80
100
120
0
100
200
300
400
500
600
700
